# Update on Ebola Treatment Center Costs and Sustainability, United States, 2019

**DOI:** 10.3201/eid2605.191245

**Published:** 2020-05

**Authors:** Jocelyn J. Herstein, Aurora B. Le, Lily A. McNulty, Sean A. Buehler, Paul D. Biddinger, Angela L. Hewlett, John J. Lowe, Shawn G. Gibbs

**Affiliations:** University of Nebraska Medical Center, Omaha, Nebraska, USA (J.J. Herstein, A.L. Hewlett, J.J. Lowe);; Indiana University, Bloomington, Indiana, USA (A.B. Le, L.A. McNulty, S.A. Buehler, S.G. Gibbs);; Massachusetts General Hospital, Boston, Massachusetts, USA (P.D. Biddinger);; Harvard Medical School, Boston (P.D. Biddinger)

**Keywords:** Ebola, Ebola virus disease, Ebolavirus, viruses, communicable diseases, emerging, hospitals, isolation, Ebola treatment centers, costs, sustainability, zoonoses, United States

## Abstract

We surveyed 56 Ebola treatment centers (ETCs) in the United States and identified costs incurred since 2014 ($1.76 million/ETC) and sustainability strategies. ETCs reported heavy reliance on federal funding. It is uncertain if, or for how long, ETCs can maintain capabilities should federal funding expire in 2020.

In 2014, a tiered network of facilities to manage patients with Ebola virus disease (EVD) was established in the United States (*1*). The Centers for Disease Control and Prevention designated 56 hospitals as Ebola treatment centers (ETCs), each equipped with specified capabilities to provide safe high-level isolation care for patients with EVD. This network was enhanced with the later designation of 10 regional Ebola and other special pathogen treatment centers (RESPTCs) with enhanced capabilities to care for patients with other highly hazardous communicable diseases (HHCDs). Since that time, efforts have been made to expand existing ETC capabilities beyond EVD in preparation for treating the next HHCD outbreak.

Previous assessments of these 56 ETCs by our team found average costs incurred to train teams, enhance physical infrastructure, and acquire advanced resources totaled nearly $1.2 million/facility (*2*). Despite these major investments, only 15–18 months after initially establishing their ETCs, by 2016 most hospitals reported challenges in sustaining ETC capabilities, and 3 centers reported they no longer maintained preparedness for EVD care (*3*).

Now, 3.5 years after our last ETC assessment, these specialized units face intensified threats to their sustainability because federal funding of these centers through the Hospital Preparedness Program (HPP) Ebola Preparedness and Response Activities is set to expire in 2020 (*4*). We aimed to determine whether additional costs for ETCs have incurred since our assessment in 2015, as well as to assess hospitals’ sustainability plans for maintaining capabilities after federal funding ceases.

## The Study

In April 2019, we sent a link to an electronic survey (Indiana University Institutional Review Board exemption #1903160012) by email to representatives from the 56 ETCs. Three additional email attempts were made to nonresponding ETCs through survey closing in June. We collected and analyzed data by using Qualtrics (https://www.qualtrics.com) software and exported them for descriptive statistical analyses.

A total of 37 (66%) ETCs responded. However, the ability to skip questions resulted in differing response rates within the survey. Three hospitals that had previously reported they no longer held ETC designation were still listed as ETCs and were therefore included in survey invitations. However, none responded. All but 1 of the remaining respondents reported they had maintained some degree of ETC capabilities. The 1 decommissioned ETC cited a lack of funding and diminished perceived threat of EVD as factors that led to reversion to a regular unit. The other 36 ETCs sustained their high-level isolation capabilities (Figure).

**Figure:**
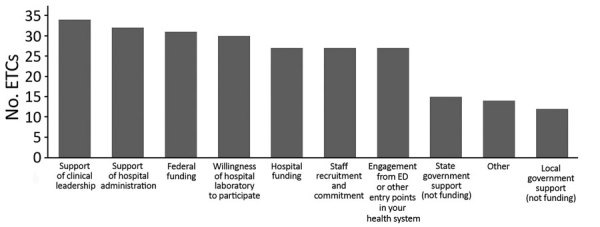
Factors that have contributed to sustaining high-level isolation capabilities among 36 ETCs, United States, 2019. Other responses that were reported by >1 ETC were a full-time-equivalent designated person responsible for management (n = 3), emergency medical services partnerships (n = 2), and support from the entire institution (n = 3). ED, emergency department; ETCs, Ebola treatment centers.

We compiled details of costs incurred by facilities since their 2014 establishment (Table). Of the 35 facilities that completed the reimbursement section, 29 (83%) reported they had received reimbursement from the federal government and 3 (9%) from the state government for costs in establishing or maintaining their unit. A total of 23 facilities reported total reimbursement amounts by the federal and state governments to date, totaling $26,546,545 (average $1,154,198/ETC). A total of 21 ETCs reported total costs and reimbursement received as of June 2019; these facilities had expended, on average, $612,664 more than their reimbursements. When we excluded federally funded RESPTCs, this figure increased to $753,015.

**Table:** Costs of establishing and maintaining Ebola treatment centers, United States, 2019*

Cost scale	Total costs	Construction/facility modifications	Administration	PPE purchases	Staff training	Laboratory equipment	Other unit purchases
Establishment†							
Average	$1,425,640	$1,029,973	NA	$166,825	$290,788	$117,134	$141,158
Median	$937,500	$561,000	NA	$87,467	$200,000	$75,000	$100,000
High	$4,650,000	$4,000,000	NA	$900,000	$1,250,000	$500,000	$450,000
Low	$100,000	$10,000	NA	$3,375	$10,000	$4,600	$4,066
Total	$45,620,489	$27,809,283	NA	$4,337,454	$7,269,711	$2,940,494	$2,964,324
Maintenance‡§							
Average	$224,664	NA	$103,151	$26,367	$70,562	$40,071	$32,679
Median	$170,000	NA	$27,500	$17,500	$45,000	$27,500	$18,000
High	$600,000	NA	$300,000	$75,000	$250,000	$224,500	$100,000
Low	$10,000	NA	$5,000	$1,500	$1,000	$500	$2,500
Total	$6,515,261	NA	$2,475,626	$685,550	$1,693,500	$561,000	$686,250
Total, establishment + maintenance¶							
Average	$1,764,922						
Median	$1,066,500						
High	$5,125,000						
Low	$140,000						
Total	$49,417,813						

*NA, not applicable; PPE, personal protective equipment. †As of June 2019, n = 32 units that provided costs to establish their facility. ‡As of June 2019, n = 29 units that provided costs to maintain their facility. §Survey tool did not differentiate between zeros and nonresponses; therefore, averages for subcategories might be inflated because zeros were considered nonresponses. ¶As of June 2019, n = 28 units that provided total costs to establish and maintain their facility.

Of the 34 ETCs that reported primary funding mechanisms for sustaining unit operations, most cited federal (n = 30, 88%) and institutional (hospital) (n = 28, 82%) funding. A total of 21 (58%) ETCs reported capabilities would be maintained after HPP funds expire in 2020; 3 (8%) reported they would no longer maintain capabilities after funding expires, and 11 (31%) were uncertain if capabilities would be maintained. Of the 21 ETCs that would maintain capabilities, nearly all reported additional funding sources would be internal (n = 17 of 18 hospitals that detailed other funding sources), and 4 explicitly noted the desire for state funding to sustain minimal capabilities and continued staff training. Of the 11 ETCs that were uncertain if capabilities would remain, all noted their commitment to sustain capabilities but voiced ambiguity as to whether their hospital would be able to provide necessary funding within their budget. When queried if ETC capabilities would be maintained once personal protective equipment (PPE) stocks expire, 27 (77%) ETCs responded yes and 8 (23%) were uncertain.

Most facilities (n = 26, 76%) used the ETC space as a functional clinical unit when not activated for HHCD care, most commonly as intensive care unit beds (n = 13). Most ETCs have been used for >1 person under investigation for EVD since June 2014 (n = 22, 63%).

## Conclusions

This assessment of ETC costs and sustainability plans is a follow-up to findings from 2015 and 2016 that surveyed the then newly established ETC network. Reported costs of establishing ETC capabilities increased by >$230,000/ETC from our spring 2015 assessment (*2*), reflecting the ongoing efforts of ETCs to prepare for EVD cases. To date, since establishment in 2014–2015, an average additional $225,000 has been spent per ETC to maintain HHCD care capabilities. Although total ETC costs have increased since our initial assessments (nearly $1.8 million compared with $1.2 million in 2015), gaps in reimbursement from federal and state funding have also increased (from $650,000 to $750,000 in non-RESPTC ETCs). Since 2016, more ETCs reported using their unit for routine use when not activated (76% vs. 58%), offsetting operational costs.

This study had limitations. ETC responses were self-reported and not validated. The survey tool defaulted nonresponses to zeros; therefore, for reported maintenance costs, averages for cost subcategories might be inflated because zeros were considered nonresponses. Since our previous assessment, 22 facilities reported new primary contacts; although we reached out to multiple persons for each ETC, it is possible personnel from nonresponding facilities have since left their position or hospital. It is also unclear how many nonresponding ETCs no longer maintain their capabilities; authors are aware of several nonresponding hospitals within the network that no longer maintain EVD care capabilities.

Since late 2015, the perceived threat of an HHCD outbreak within the borders of the United States has waned, and the perceived demand for numerous US hospitals to maintain a high level of preparedness for HHCDs has dwindled. In tandem with inadequate funding, more ETCs have elected to forgo high-level isolation capabilities. The establishment of RESPTCs sought to centralize capabilities at a regional level, but many ETCs noted that since 2014, major investments in establishing high-level isolation capabilities could prompt continued internal financial support if federal funding ceases.

However, ETCs reported heavy reliance on federal funding; nearly all reported it as their primary funding stream and leading factor in maintaining capabilities. The 2020 expiration of HPP funds threatens the existence of this network. It is unknown if—and for how long—many ETCs could maintain capabilities solely with internal financial support, or if the United States will revert to the level of HHCD preparedness before 2014. The ongoing EVD outbreak in the Democratic Republic of the Congo and the rise of 2019 novel coronavirus disease in China are reminders that HHCD outbreaks are increasingly regular occurrences. The high proportion of ETCs surveyed that have used their ETC for a person under investigation since 2014 (63%) further underscores the ongoing need of such specialized units across the country. The US healthcare system has made major strides in HHCD domestic preparedness capability since 2014. However, on the basis of study responses, the US health system could again be vulnerable and inadequately prepared for the next HHCD threat if federal HPP funding is not renewed.
